# Innate immune responses after stimulation with Toll-like receptor agonists in ex vivo microglial cultures and an in vivo model using mice with reduced microglia

**DOI:** 10.1186/s12974-021-02240-w

**Published:** 2021-09-06

**Authors:** James A. Carroll, Brent Race, Katie Williams, James F. Striebel, Bruce Chesebro

**Affiliations:** Laboratory of Persistent Viral Diseases, Rocky Mountain Laboratories, National Institute of Allergy and Infectious Diseases, National Institutes of Health, 903 South Fourth Street, Hamilton, MT 59840 USA

**Keywords:** Microglia, Neuroinflammation, Innate immunity, PLX5622, CSF1R, Toll-like receptor, LPS, Imiquimod, CpG-ODN

## Abstract

**Background:**

Past experiments studying innate immunity in the central nervous system (CNS) utilized microglia obtained from neonatal mouse brain, which differ developmentally from adult microglia. These differences might impact our current understanding of the role of microglia in CNS development, function, and disease.

**Methods:**

Cytokine protein secretion was compared in ex vivo P3 and adult microglial cultures after exposure to agonists for three different toll-like receptors (TLR4, lipopolysaccharide [LPS]; TLR7, imiquimod [IMQ]; and TLR9, CpG Oligodeoxynucleotide [CpG-ODN] 1585). In addition, changes in inflammatory gene expression in ex vivo adult microglia in response to the TLR agonists was assessed. Furthermore, in vivo experiments evaluated changes in gene expression associated with inflammation and TLR signaling in brains of mice with or without treatment with PLX5622 to reduce microglia.

**Results:**

Ex vivo adult and P3 microglia increased cytokine secretion when exposed to TLR4 agonist LPS and to TLR7 agonist IMQ. However, adult microglia decreased expression of numerous genes after exposure to TLR 9 agonist CpG-ODN 1585. In contrast, in vivo studies indicated a core group of inflammatory and TLR signaling genes increased when each of the TLR agonists was introduced into the CNS. Reducing microglia in the brain led to decreased expression of various inflammatory and TLR signaling genes. Mice with reduced microglia showed extreme impairment in upregulation of genes after exposure to TLR7 agonist IMQ.

**Conclusions:**

Cultured adult microglia were more reactive than P3 microglia to LPS or IMQ exposure. In vivo results indicated microglial influences on neuroinflammation were agonist specific, with responses to TLR7 agonist IMQ more dysregulated in mice with reduced microglia. Thus, TLR7-mediated innate immune responses in the CNS appeared more dependent on the presence of microglia. Furthermore, partial responses to TLR4 and TLR9 agonists in mice with reduced microglia suggested other cell types in the CNS can compensate for their absence.

**Supplementary Information:**

The online version contains supplementary material available at 10.1186/s12974-021-02240-w.

## Background

Microglia are self-renewing immune-like glia cells, which are derived from erythro-myeloid progenitors in the yolk sac that migrate to colonize the central nervous system (CNS) rudiment [1, 2]. These cells are important in host defense against infection and in responding to cellular damage in the CNS. Furthermore, they play diverse roles in the CNS that include neurodevelopment, phagocytosing dead or dying cells, and maintenance of homeostasis in the brain [3–5]. Microglia also influence astrocytes in the CNS in response to lipopolysaccharide (LPS) by secretion of immune effectors (TNFα, IL-1α, and C1q) that induce astrocytes to assume a proinflammatory phenotype, termed A1-astrocytes [6, 7]. Thus, microglia can directly and indirectly influence neuroinflammation and neurodegeneration.

As the primary immune cell in the CNS, microglia are important in innate immunity, expressing numerous scavenger receptors [8], and all the Toll-like receptors (TLR) [9, 10] that can detect pathogen-associated and damage/danger-associated molecular patterns (PAMPs and DAMPs) [10–12]. For example, TLR4 can interact with PAMPs such as bacterial LPS, mannuronic acid polymers, teichuronic acid, and the F protein of respiratory syncytial virus (reviewed in [13, 14]). TLR4 also recognizes endogenous ligands and DAMPs such as different heat-shock proteins, HMGB1, S100 proteins, fibronectin, and CD138. The dual functionality of this class of receptors appears to be widespread and shared among other TLRs (reviewed in [15, 16]).

Studying the influence of microglia within the CNS became more achievable with the development of compounds that chemically reduce their numbers. Microglia rely on continued signaling through the CSF-1 receptor (CSF-1R) for their survival [17–19]. Loss or reduction of CSF-1R signaling can have multiple effects ranging from a non-proliferative state [20, 21] to a reduction in microglia cell numbers throughout the CNS [18] and the eye [22, 23]. Use of small molecules like CSF-1R inhibitors PLX3393 and PLX5622 (Plexxicon, Berkeley, CA) eliminate most microglia in the CNS by apoptosis, resulting in an 80% to 90% reduction in microglia after 7 days of treatment [18, 24].

Our laboratory became interested in microglia and their influence through studies of gliosis and CNS inflammation in response to prion infection in mice [25–28]. We recently reported the importance of microglia in host defense during prion infection [29], and that the expression pattern of microglial genes in the prion-infected brain contrasts with those expressed in other neurodegenerative disease models [30]. Furthermore, we have isolated postnatal [26] and adult microglia [31] for ex vivo studies to better determine the direct response of these cells to various prion-related conditions.

Similar to our earlier studies, most past experiments using primary microglia to study innate immunity were performed using neonatal microglia or immortalized BV2 cells that are microglia-like [32–38], but recent findings indicate that these cells are different from adult microglia [39, 40]. Butovsky et al. indicate that the gene expression patterns of freshly isolated newborn microglia, primary cultured newborn microglia, and cultured microglial cell lines differ greatly from those identified in freshly isolated adult microglia. Moreover, they discovered that addition of TGF-β to the culture medium allowed primary cultured adult microglia to retain a homeostatic microglial expression signature like that of freshly isolated adult cells [39]. With this in mind, we began a series of studies to contrast the responses of neonatal and adult microglia to various TLR agonists. Furthermore, we used PLX5622 to ablate microglia from the CNS to evaluate the contribution of microglia to inflammation and TLR signaling in the complex milieu of the brain in response to TLR4, TLR7, and TLR9 agonists.

## Methods

### Microglia isolation and culture

#### From adult mice

Microglia were isolated from 6 to 8-week-old C57BL/10 mice. Mice were euthanized then transcardially perfused with 10.0 ml sterile PBS. Each brain was removed and diced into 2 mm pieces with a sterile scalpel and further dissociated using the Neural Tissue Dissociation Kit – Postnatal Neurons (catalog # 130-094-802) in combination with the gentle magnetic-activated cell separation (MACS) dissociator for automated tissue separation into a single-cell suspension (Miltenyi Biotec). Tissue dissociation was carried out per the manufacturer’s instructions.

The single-cell suspension for each brain was strained through a 70 μm MACS SmartStrainer (Miltenyi Biotec), centrifuged (300×*g*, 23 °C, 10 min), and suspended in 1.8 ml sterile phosphate-buffered saline (PBS, Gibco), pH 7.2 supplemented with 0.5% bovine serum albumin (PBS-BSA). Myelin contamination was reduced by the addition of 200 μl Myelin Isolation Beads (Miltenyi, product # 130-104-253) to the cell suspension and incubated for 15 min at 4 °C. The volume was then adjusted to 3.0 ml with PBS-BSA and subjected to magnetic activated cell separation (MACS) by adding 1.0 ml to three LS Columns (Miltenyi Biotec). The flow-through was collected (unlabeled cell suspension), and each column was washed twice with 1.0 ml of PBS-BSA. All washes were then combined with the collected flow-through, yielding a total of approximately 9.0 ml of a myelin-reduced cell suspension for each brain. The myelin-reduced single-cell suspension was then centrifuged (300×*g*, 23 °C, 10 min) and cells were suspended in 180 μl PBS-BSA for microglia isolation.

Microglia from a single mouse brain were labeled in the single-cell suspension by adding 20 μl CD11b MicroBeads (product # 130-093-634, Miltenyi Biotec) with incubation for 15 min at 4 °C. The volume was then increased to 5.0 ml with PBS-BSA and cells were pelleted by centrifugation (300×*g*, 23 °C, 10 min). The washed cells were suspended in a final volume of 1.0 ml PBS-BSA, applied to a LS Column, and labeled microglia were isolated by MACS. The column was rinsed 3 times with 3.0 ml PBS-BSA, where the flow-through was discarded. The LS Column containing isolated microglia was removed from the magnetic field, 5.0 ml PBS-BSA was added to the column, and microglia were flushed into a fresh sterile tube using the supplied plunger. We typically acquire approximately 4 × 10^5^ highly purified microglia per adult mouse brain (> 99% IBA positive cells) using this method of isolation [31].

#### From postnatal mice

Mixed glial cells from cerebral cortices of 3-day-old mice were cultured as a primary passage in T-75 flasks for 10–14 days as described previously [25]. To harvest microglia, flasks were rotated at 200 rpm using an orbital shaker for 2 h at 37 °C. Cell suspensions were centrifuged at 300×*g*, resuspended in 180 μl PBS-BSA, and labeled in the single-cell suspension by adding 20 μl CD11b MicroBeads. Postnatal microglia were isolated by MACS as indicated above, with > 99% IBA-positive cells in the final culture.

Purified postnatal and adult microglia were centrifuged (300×*g*, 23 °C, 10 min) and suspended in DMEM/F-12 Glutamax (Invitrogen) supplemented with 10% fetal calf serum (FCS), 100 U/ml penicillin, 100 ng/ml streptomycin, 10 ng/ml mouse recombinant carrier-free MCSF (CSF-1, R&D Systems), and 50 ng/ml human recombinant TGFβ1 (Miltenyi Biotec) [39]. Microglia were then transferred into 25 cm^2^ Ultra-Low Attachment polystyrene flasks (Corning) and incubated at 37 °C with 5.0% CO_2_. The medium was changed every 5 days with fresh medium. Microglia were cultured 10 days before harvesting for experimental studies.

Prior to experimentation, a portion of each microglial preparation was assayed for purity by immunofluorescent microscopy using antibody to IBA-1. Briefly, cells attached to chamber slides were permeabilized with PBS with 0.1% Triton X-100 for 15 min. Samples were primarily blocked for 30 min in 0.1 M glycine and then secondarily blocked in 2.0% normal donkey serum, 1.0% bovine serum albumin, 0.1% Triton X-100, and 0.05% Tween 20 in PBS for an hour. Slides were exposed to rabbit anti-Iba1 (Wako, Richmond, VA, USA) (dilution 1:1000) for 1 h at room temperature, followed by two washes in PBS with 1% Triton X-100. Slides were then probed with an Alexa Fluor 488 dye conjugated anti-rabbit antibody (Invitrogen) at a dilution of 1:500 for an hour at room temperature. Slides were washed 3 times with PBS with 0.1% Triton X-100 for 15 min each. ProLong Gold antifade mountant with DAPI (Invitrogen) was applied to each slide prior to coverslipping. IBA-1 positive cells were then manually counted by randomly selecting 16 microscopy fields of view that totaled over 5000 cells. With this technique, our IBA-1-positive cells in our microglia preparations averaged 99.69%.

### Cultured microglia exposure to TLR agonists

Purified postnatal (P3) and adult microglia were counted using the Muse Count and Viability Kit (Millipore-Sigma) with the Muse flow cytometer (Millipore-Sigma) and seeded at 1.0 × 10^4^ cells per well into 24-well Ultra-Low Attachment polystyrene plates (Corning) in 500 μl of DMEM/F-12 Glutamax (Invitrogen) supplemented with 10% FCS, 100 U/ml penicillin, 100 ng/ml streptomycin, and 10 ng/ml mouse recombinant carrier-free MCSF (CSF-1, R&D Systems) and allowed to recover for 24 h. Microglia were then exposed to the ultra-pure LPS (TLR4 agonist L3024, Sigma-Aldrich) at a final concentration of 1.0 μg/ml, Imiquimod (TLR7 agonist R837, InvivoGen) at a final concentration of 5.0 μM, CpG oligonucleotides (CpG-ODN) 1585 (TLR9 agonist, InvivoGen) at a final concentration of 80 nM, or the medium alone as a control. These concentrations have been used previously with reliable results [25, 33]. Microglia were incubated for 10 h, at which time spent medium was stored at − 20 °C and/or total RNA from cells was isolated from adult microglia cultures for further analysis. A 10-h exposure to TLR agonists was chosen to reduce excessive microglia overactivation induced apoptosis [41], thus allowing more accurate analysis of adult microglia changes in transcription.

### PLX5622 treatment of mice

C57BL/10 (C57) mice were originally obtained from Jackson Laboratories and have been inbred at RML for several years. Six to eight-week-old male and female mice were fed purified rodent diet AIN-76A (D10001, Research Diets, Inc.) with or without supplementation with compound PLX5622 (1200 ppm chow, kindly provided by Plexxikon Inc., Berkeley, CA). Mice fed this concentration of PLX5622 in rodent chow has been shown to reduce microglia in the brain by approximately 80% within 7 days and approximately 90% within 21 days of administration [24]. Mice either untreated or treated with PLX5622 for approximately 28 days were exposed to TLR agonists by intraventricular injection of 9.0 μl LPS (0.28 μg/μl in PBS, final of 2.5 μg), Imiquimod (IMQ) (5.6 μg/μl in PBS, final of 50 μg), or CpG-ODN 1585 (11.1 μg/μl in PBS, final of 100 μg) or an equal volume of PBS as a control. These concentrations have been show in previous studies to be effective at eliciting an innate immune response in the CNS of mice [32, 42]. Intraventricular injections were performed using a stereotactic pump at − 0.3 bregma, 1 mm lateral, and 2.5 mm deep. To minimize reflux, the inoculum was introduced slowly at a rate of ~ 2.0 μl per second, and the needle was then withdrawn after one-minute post inoculation. After 6 h of exposure, injected mice were euthanized by isoflurane anesthesia overdose followed by cervical dislocation. This 6-h time point was chosen to minimize the influence of infiltrating peripheral leukocytes on CNS innate immune responses [43, 44]. Brains were removed and half was used for RNA isolation and analysis. Brain portions for histochemical analysis were immersed in 10% neutral-buffered formalin (3.7% formaldehyde) for histology.

### Immunohistochemical staining of microglia

Portions of brain were removed and placed in 10% neutral-buffered formalin for 3 to 5 days. Tissues were then processed and embedded in paraffin. Sections were cut using a standard Leica microtome, placed on positively charged glass slides, and air-dried overnight at room temperature. The following day slides were heated in an oven at 60 °C for 30 min.

Deparaffinization, antigen retrieval, and staining were performed using the Ventana automated Discovery XT stainer. To stain microglia, rabbit anti-Iba1 was used at a 1:2000 dilution and was a gift from Dr. John Portis, RML, Hamilton, MT. Primary antibodies were diluted in PBS containing stabilizing protein and 0.1% Proclin 300 (Ventana Antibody Dilution Buffer). Diluent without antibody was used as a negative control. Ventana streptavidin-biotin alkaline phosphatase system was used to detect Iba1 [45] with the exceptions that the secondary antibody was goat anti-rabbit Ig (Biogenex, HK336-9R) and Fast Red chromogen was used. Hematoxylin was used as a counterstain for all slides. Slides were examined and photomicrographs were taken and observed using an Olympus BX51 microscope and Microsuite FIVE software. Comparisons of the number of Iba1-positive microglia between PLX5622-treated and untreated cohorts were compared by unpaired Student’s *t* test using GraphPad Prism 8 as described previously [29].

### RNA isolation

Total RNA was isolated from the left hemisphere of mouse brain by dissociation of tissue in 2 ml TRI-reagent (Sigma) following the manufacturer’s protocol. Isolated RNA was rinsed in 2 ml 75% ethanol, centrifuged for 10 min at 13,000×*g*, and air dried. Total RNA was suspended in 100 μl of DNase reaction buffer (Ambion) and digested with 6 units of DNase I (Ambion) of 30 min at room temperature. RNA was re-isolated and cleaned using RNA Clean & Concentrator-25 column kit (Zymo Research), eluted with 150 μl nuclease-free water with 1× RNase Inhibitor (SUPERase-In, Ambion), and stored at − 80 °C until use.

RNA from adult microglia was isolated by Quick-RNA MiniPrep (Zymo Research). Briefly, 150 μl of RNA lysis buffer was added to each well for 5 min. An equal volume of 100% ethanol was added to each sample and mixed by pipetting. The mixture was added to the provided Zymo-spin columns and RNA was isolated and washed per the manufacturer’s instructions. RNA was eluted from the columns by the addition of 50 μl nuclease-free water (Ambion), DNased with 2 units of DNase I (Ambion) of 30 min at room temperature, re-isolated and cleaned using RNA Clean & Concentrator-25 column kit (Zymo Research), and eluted with 15 μl with nuclease-free water supplemented 1× SUPERase-In RNase inhibitor and stored at − 80 °C.

### qRT-PCR analysis

For quantitative analysis of changes in transcription from brain tissue using qRT-PCR arrays, 400 ng of high-quality RNA from each sample was reverse transcribed to synthesize cDNA using the RT^2^ First Stand Kit per manufacturer’s instructions (QIAGEN). For quantitative analysis in transcription from adult microglia, 10 ng of RNA from each culture well was pre-amplified using the RT^2^ PreAMP cDNA synthesis kit (330451) with accompanying RT^2^ PreAMP Mouse Inflammatory Cytokine & Receptors Pathway Primer Mix (330241 PBM-011Z) according to instructions by QIAGEN. Each cDNA reaction was mixed with 2x RT2 SYBR Green Mastermix purchased from QIAGEN with RNase-free water to a final volume of 1.3 ml. Ten microliters of the mixture was then added to each well of a 384-well format plate of either the Mouse Inflammatory Cytokine & Receptors super array PAMM-011ZE or Mouse Toll-Like Receptor Signaling super array PAMM-018ZE (QIAGEN).

The analysis was carried out on an Applied Biosystems ViiA 7 Real-Time PCR System with a 384-well block using the following conditions: 1 cycle at 10 min, 95 °C; 40 cycles at 15 s, 95 °C then 1 min, 60 °C with fluorescence data collection. Melting curves were generated at the end of the completed run to determine the quality of the reaction products. Raw threshold cycle (C_T_) data was collected with a C_T_ of 35 as the cutoff. C_T_ data was analyzed using the web-based RT^2^ Profiler PCR Array Data Analysis from QIAGEN. All C_T_ values were normalized to the average of the C_T_ values for the housekeeping genes *Actb*, *Gapdh*, and *Hsp90ab1*. Changes in transcription were calculated by the software using the ΔΔC_T_ based method [46]. Statistical analysis was performed using the unpaired Student’s *t* test to compare the replicate ΔC_T_ values for each gene in the control group versus infected groups [47]. A mean of ≥ or ≤ 2.0-fold change and *P* value of ≤ 0.05 were considered significant. For qRT-PCR data, we did not adjust *P* values for multiple comparisons since we were interested in only controlling for the individual error rate, where an adjustment for multiple tests is deemed unnecessary. Venn diagrams and heatmap hierarchical cluster analysis of the full datasets were composed using InteractiVenn [48] and Heatmapper [49], respectively.

### Cytokine and chemokine quantification

Fifty-microliters of medium from adult microglia incubated with various TLR agonists or medium alone was used to quantify cytokine and chemokine secretion. Levels of 23 immune effectors (Mouse 23-plex panel group 1, M60009RDPD) or 33 immune effectors (Mouse Chemokine Panel 33-Plex #12002231) were analyzed using the Bio-Plex suspension array system in accordance with the manufacturer’s instructions (Bio-Rad). Graphing and statistical analysis were performed using a one-way ANOVA with an uncorrected Fisher’s least significant difference test (GraphPad Prism 8) either to compare P3 to adult microglia exposed to TLR agonists or to compare adult microglia exposed to medium alone relative to those incubated with TLR agonists, with a *P* value ≤ 0.05 considered significant.

## Results

### Neonatal and adult microglia responses to TLR agonists

We and others have previously experimented with neonatal cells to determine microglial responses because of their relative ease of isolation, but recent advancements in techniques such as magnetic activated cell separation have made isolation of adult mouse microglia from the brain more feasible. Because neonatal and adult microglia demonstrate noticeable differences in their homeostatic gene expression signatures [39], we were interested in determining if postnatal (P3) and adult microglia respond similarly to TLR ligands following culture conditions that retain a homeostatic phenotype.

**Fig. 1:**
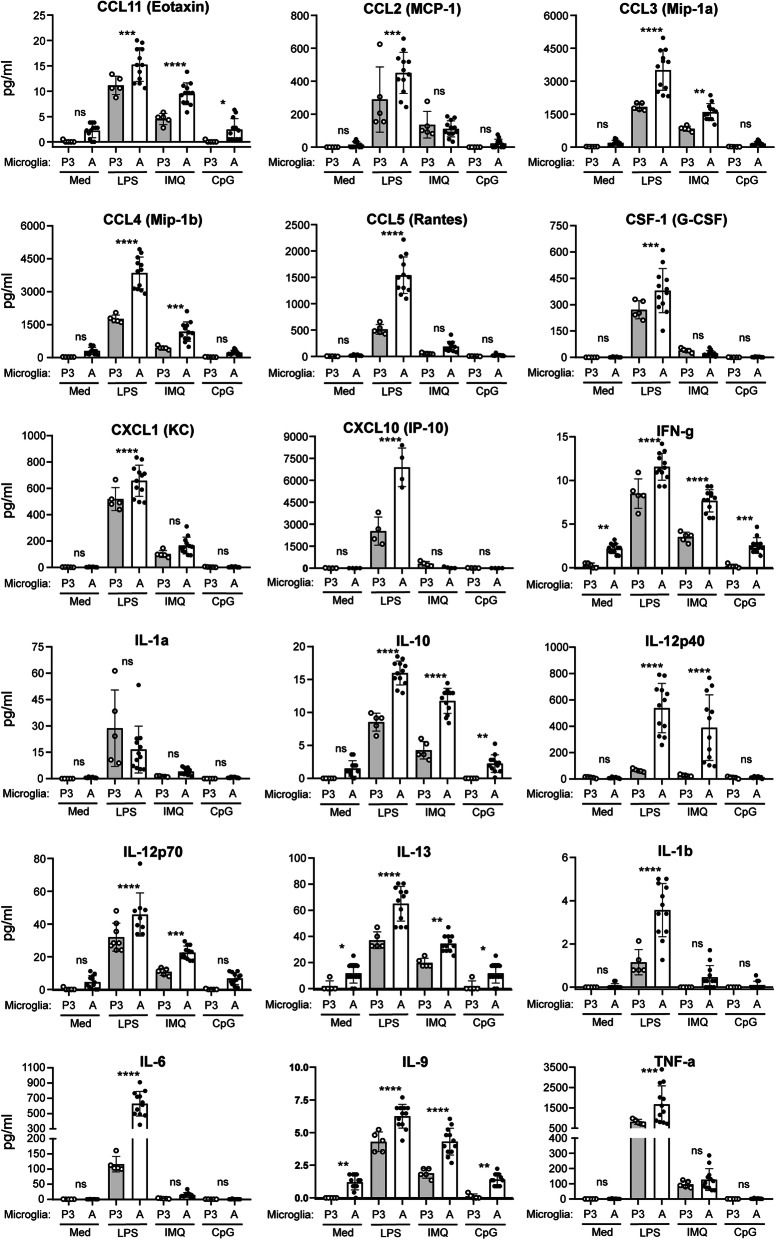
Comparison of immune effector secretion by postnatal and adult microglia when exposed to TLR agonists ex vivo. Equal numbers of microglia isolated from postnatal (P3) and adult (A) mice were exposed to LPS, IMQ, CpG-ODN 1585 (CpG), or medium alone (Med) for 10 h before harvesting. Secreted immune effectors were measured in the harvested medium by Bio-Plex suspension array system. Each dot indicates an individual well of microglia that was isolated from either 6 (adults) or 4 (P3) mice and cultured separately. Statistical analysis was performed using a one-way ANOVA with an uncorrected Fisher’s least significant difference test to compare P3 to adult microglia exposed to TLR agonists. ****P ≤ 0.0001; ***P ≤ 0.001; **P ≤ 0.01; *P ≤ 0.05. *ns* not significant

Identically seeded postnatal (P3) and adult microglia were exposed for 10 h to TLR4 agonist LPS, the TLR7 agonist Imiquimod (IMQ), or the TLR9 agonist class A CpG oligonucleotides (CpG-ODN) 1585. Medium was collected and immune effector release was determined, with comparisons presented in Fig. 1. LPS induced cytokine/chemokine production in all samples tested, but levels were statistically higher in adult microglia relative to P3 cells for most immune effectors. Only IL-1α release from LPS-induced P3 and adult microglia were similar. IMQ also induced higher levels of many immune effectors (i.e., CCL3, CCL4, and IL-12p40) in adult microglia cultures compared with postnatal, but equivalent amounts of TNF-α were secreted by P3 and adult microglia (Fig. 1).

CpG-ODN 1585 was a weak agonist under our culture conditions and did not induce any detectable changes in secretion of immune effectors above our media-only baseline (Fig. 1). These results strengthened past studies where the class A CpG-ODN 1585 did not induce a neuroprotective phenotype in cultured microglia, though classes B and C could [50]. Additionally, there were a small number of effectors that were undetectable in any sample regardless of cell type or agonist (Table 1).

**Table 1:** Immune effectors below the level of detection in medium of P3 or adult microglia after TLR agonist LPS, IMQ, or CpG-ODN 1585 exposure

IL-2	IL-13
IL-3	IL-17 A
IL-4	CCL11 (Eotaxin)
IL-5	GM-CSF (CSF2)
IL-9	

Thus, our initial comparison identified several differences between adult and P3 microglia. First, the baseline for several of the cytokines tested (IFN-g, IL-13, and IL-9) was significantly higher in adult relative to P3 microglia. Second, in most instances, the magnitude of inflammatory response was greater in adult microglia when exposed to LPS and IMQ ex vivo. Lastly, the class A CpG-ODN 1585, unlike the class B agonist CpG-ODN 1826 [33], is a poor TLR9 agonist for cultured microglia.

### Expression and secretion of additional inflammatory effectors in adult microglia in response to TLR agonists

**Fig. 2:**
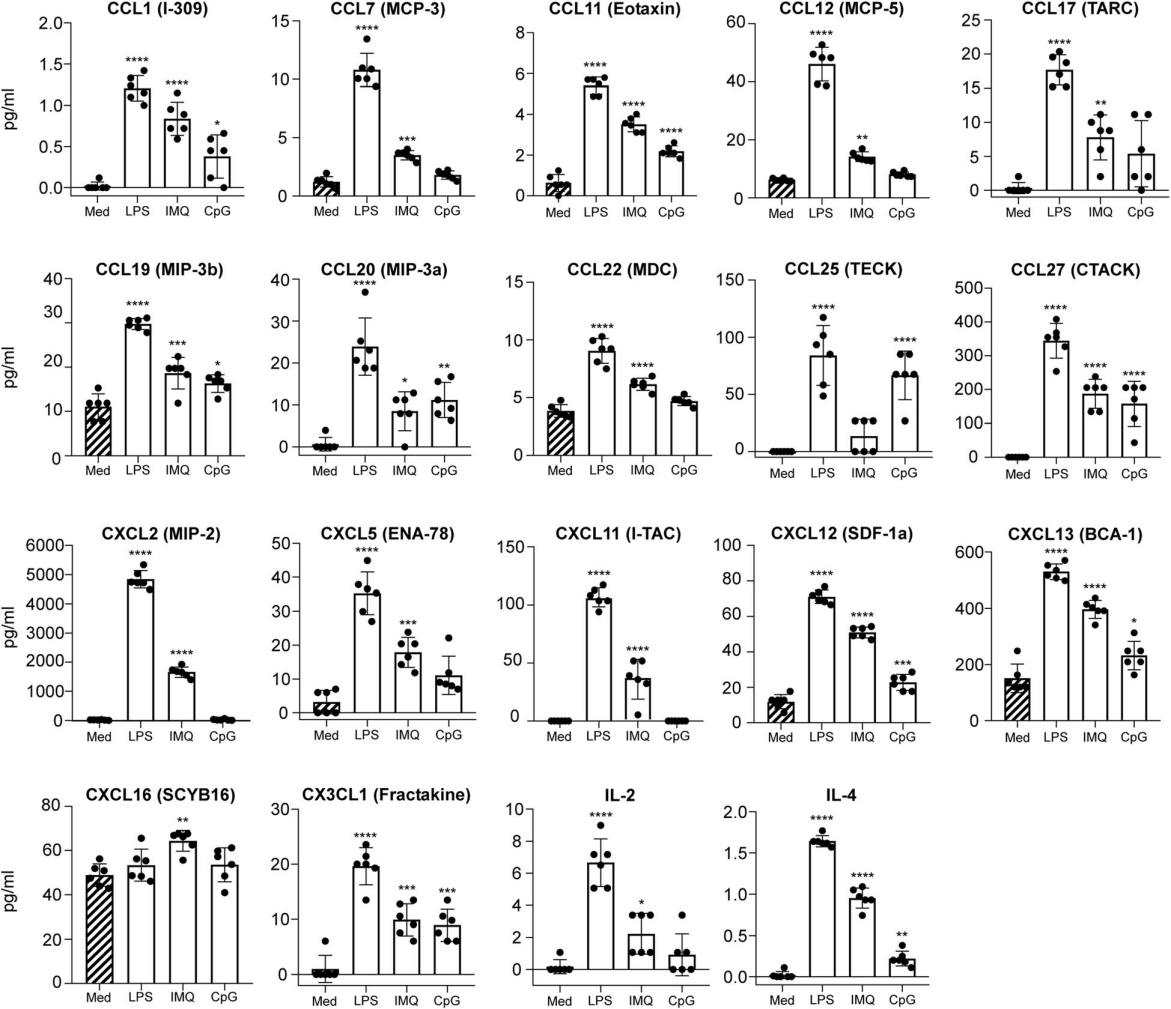
Additional characterization of immune effector secretion by adult microglia when exposed to TLR agonists ex vivo. Adult microglia were exposed to LPS, IMQ, CpG-ODN 1585 (CpG), or medium alone (Med) for 10 h before harvesting. Secreted immune effectors were measured in the harvested medium by Bio-Plex suspension array system. Each dot indicates an individual well of microglia that was isolated from 3 adult mice and cultured separately. Statistical analysis was performed using a one-way ANOVA with an uncorrected Fisher’s least significant difference test to compare P3 to adult microglia exposed to TLR agonists. ****P ≤ 0.0001; ***P ≤ 0.001; **P ≤ 0.01; *P ≤ 0.05

Adult microglia are more developed than those isolated from neonatal mice and express several TLR and scavenging receptors that are decreased in neonatal microglia [39]. Because the innate immune response of adult microglia is not as well understood as that of neonates, we wanted to further characterize the innate immune responses of homeostatic adult microglia exposed to TLR agonists ex vivo. Thus, we evaluated the secretion of additional immune effectors that are less commonly assayed (Fig. 2). Many chemokines were elevated with LPS stimulation of adult microglia. Furthermore, the exposure of adult microglia to IMQ and CpG-ODN 1585 also increased secretion of several effectors albeit typically at lower levels than LPS.

**Fig. 3:**
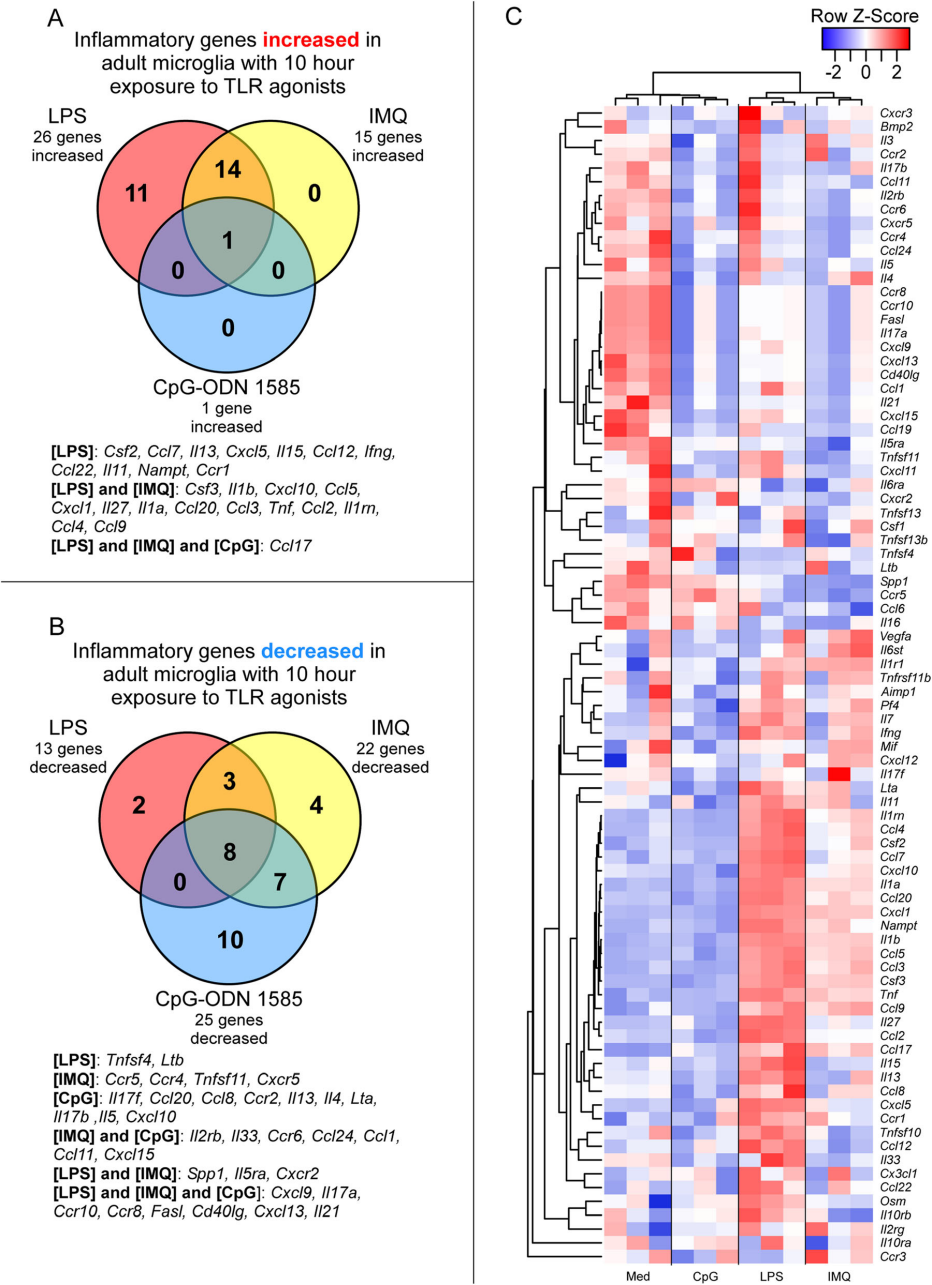
Alterations in expression of 84 inflammation-associated genes in adult microglia after 10 h of exposure to TLR agonists ex vivo. RNA from cultured microglia isolated from 3 adult mice and exposed to LPS, IMQ, CpG-ODN 1585 (CpG), or medium alone (Med) was assayed by qRT-PCR using the mouse inflammatory cytokine and receptor array by QIAGEN. Panel **A** shows a Venn diagram of inflammatory genes increased in adult microglia after TLR agonists exposure relative to adult microglia in medium alone. Panel **B** shows a Venn diagram of inflammatory genes decreased in adult microglia in response to TLR agonists relative to adult microglia in medium alone. Panel **C** is a Pearson correlation heatmap and average linkage hierarchical cluster analysis of the row Z-scores of the Delta C_T_ from the array of 84 inflammatory cytokine and receptor genes obtained from the analysis of RNA from adult microglia exposed to TLR agonists and medium alone. Each group consisted of an *n* = 3. Refer to Additional File 1 for the complete dataset

Next, we quantified changes in adult microglia gene expression in response to LPS, IMQ, and CpG-ODN 1585 using a qRT-PCR array targeting 84 mouse inflammatory cytokine and receptor genes (refer to Additional file 1 for complete dataset). Comparing changes in transcription of adult microglia exposed to TLR agonists relative to medium alone, the greatest number of inflammatory genes were increased in expression when adult microglia were exposed to LPS (Fig. 3A and Table 2). Exposure to IMQ increased expression of 15 genes, with most shared with LPS exposure but at lower transcript levels (Fig. 3A and Table 2). CpG-ODN 1585 increased the expression of just a single inflammatory gene (*Ccl17*) included on this array (Fig. 3A and Table 2).

**Table 2:** Changes in gene expression in adult microglia exposed to TLR agonists for 10 h relative to unexposed adult microglia in medium alone

Gene	LPS (*n* = 3)		IMQ (*n* = 3)		CpG-ODN 1585 (*n* = 3)	
	FC	*P* value	FC	*P* value	FC	*P* value
*Ccl1*	− 1.7	3.0E-01	− **4.6**	**7.3E-03**	− **4.4**	**1.6E-02**
*Ccl11*	− 1.2	8.5E-01	− **5.3**	**1.8E-02**	− **5.2**	**3.1E-02**
*Ccl12*	**13.0**	**4.5E-03**	− 2.0	2.4E-01	1.2	7.6E-01
*Ccl17*	**76.1**	**6.8E-03**	**25.4**	**9.6E-04**	**4.7**	**3.6E-02**
*Ccl2*	**85.9**	**1.4E-04**	**2.6**	**7.3E-03**	− 2.0	2.2E-01
*Ccl20*	**330.2**	**3.5E-05**	**15.7**	**2.0E-03**	− **3.3**	**2.1E-02**
*Ccl22*	**7.8**	**6.6E-02**	1.6	6.3E-01	1.3	8.0E-01
*Ccl24*	− 2.0	1.9E-01	− **4.2**	**1.2E-02**	− **3.6**	**2.9E-02**
*Ccl3*	**155.5**	**3.9E-04**	**22.2**	**4.1E-03**	− 1.5	2.9E-01
*Ccl4*	**48.8**	**1.7E-03**	**4.4**	**4.7E-02**	− 1.6	2.7E-01
*Ccl5*	**1671.6**	**6.9E-05**	**127.7**	**2.9E-04**	− 1.3	7.9E-01
*Ccl7*	**58.0**	**9.0E-04**	2.7	1.7E-01	− 1.0	9.8E-01
*Ccl8*	4.3	1.2E-01	-1.2	8.1E-01	− **3.3**	**2.1E-02**
*Ccl9*	**24.3**	**1.2E-02**	**13.7**	**1.9E-02**	− 1.1	8.8E-01
*Ccr1*	**2.7**	**2.3E-02**	1.6	2.6E-01	1.4	4.9E-01
*Ccr10*	− **2.3**	**1.0E-04**	− **3.5**	**7.3E-03**	− **3.3**	**2.1E-02**
*Ccr2*	− 1.3	7.1E-01	− 1.6	5.7E-01	− **3.3**	**2.1E-02**
*Ccr4*	− 2.0	3.6E-01	− **5.1**	**2.9E-02**	− 3.3	7.9E-02
*Ccr5*	− 2.5	9.1E-02	− **4.5**	**7.1E-03**	1.2	5.9E-01
*Ccr6*	− 1.2	8.3E-01	− **3.8**	**7.1E-03**	− **3.2**	**1.2E-02**
*Ccr8*	− **2.3**	**1.0E-04**	− **3.5**	**7.3E-03**	− **3.3**	**2.1E-02**
*Cd40lg*	− **2.7**	**2.0E-03**	− **3.9**	**7.7E-03**	− **3.8**	**1.9E-02**
*Csf2*	**211.5**	**1.1E-03**	8.9	6.8E-02	− 1.0	9.7E-01
*Csf3*	**13,079.3**	**3.0E-05**	**553.8**	**1.8E-06**	− 1.8	4.2E-01
*Cxcl1*	**1,668.9**	**9.1E-06**	**160.9**	**1.2E-05**	− 1.9	2.2E-01
*Cxcl10*	**2,193.5**	**7.7E-05**	**10.7**	**3.4E-02**	− **19.3**	**3.6E-02**
*Cxcl13*	− **2.9**	**9.8E-03**	− **4.4**	**1.2E-02**	− **4.2**	**2.3E-02**
*Cxcl15*	− 2.4	1.7E-01	− **6.1**	**3.3E-02**	− **6.9**	**1.9E-02**
*Cxcl5*	**30.3**	**4.7E-05**	2.1	1.4E-01	1.1	9.1E-01
*Cxcl9*	− **2.1**	**4.9E-03**	− **3.5**	**7.3E-03**	− **3.3**	**2.1E-02**
*Cxcr2*	− **3.6**	**9.8E-02**	− **6.0**	**4.8E-02**	− 2.1	5.4E-01
*Cxcr5*	1.0	9.8E-01	− **9.6**	**2.1E-02**	− 4.3	1.8E-01
*Fasl*	− **2.3**	**1.0E-04**	− **3.5**	**7.3E-03**	− **3.3**	**2.1E-02**
*Ifng*	**11.0**	**4.0E-02**	1.2	8.7E-01	− 6.1	6.7E-02
*Il11*	**7.2**	**3.3E-02**	1.8	6.1E-01	− 1.7	6.5E-01
*Il13*	**31.4**	**6.4E-04**	− 1.3	8.2E-01	− **3.3**	**2.1E-02**
*Il15*	**16.1**	**3.1E-03**	− 2.1	9.7E-02	− 1.8	1.5E-01
*Il17a*	− **2.2**	**1.8E-03**	− **3.5**	**7.3E-03**	− **3.4**	**2.1E-02**
*Il17b*	− 1.8	7.1E-01	− 5.9	1.3E-01	− **9.6**	**3.0E-02**
*Il17f*	− 1.5	1.4E-01	1.2	8.9E-01	− **3.3**	**1.9E-02**
*Il1a*	**595.2**	**2.9E-05**	**34.2**	**7.2E-05**	− 1.4	3.9E-01
*Il1b*	**5258.1**	**1.9E-05**	**248.9**	**1.1E-04**	− 1.3	7.1E-01
*Il1rn*	**52.8**	**9.3E-04**	**6.3**	**1.4E-02**	− 1.5	2.7E-01
*Il21*	− **4.0**	**5.0E-02**	− **6.0**	**3.3E-02**	− **5.7**	**4.3E-02**
*Il27*	**1463.6**	**4.7E-06**	**5.8**	**3.6E-02**	1.3	8.6E-01
*Il2rb*	− 1.4	6.2E-01	− **3.5**	**7.3E-03**	− **3.3**	**2.1E-02**
*Il33*	2.0	4.3E-01	− **3.5**	**7.3E-03**	− **3.3**	**2.1E-02**
*Il4*	− 1.7	1.8E-01	− 1.6	3.5E-01	− **3.3**	**2.1E-02**
*Il5*	− 2.0	5.2E-01	− 6.7	5.4E-02	− **10.6**	**2.9E-02**
*Il5ra*	− **2.2**	**2.5E-04**	− **3.5**	**7.3E-03**	− 1.8	1.6E-03
*Nampt*	4.6	1.1E-01	1.2	8.3E-01	− **4.8**	**2.7E-02**
*Spp1*	− **3.7**	**3.6E-02**	− 2.6	3.6E-01	− 3.2	1.1E-01
*Tnf*	**3.5**	**5.5E-04**	1.7	2.5E-02	− 1.0	6.7E-01
*Tnfsf11*	− **2.2**	**1.8E-02**	− **3.1**	**1.0E-03**	− 1.3	1.0E-01
*Tnfsf4*	**89.9**	**1.0E-03**	**19.0**	**2.7E-03**	1.5	4.0E-01

*n* is the number of independent microglia RNA samples tested. Fold change (FC) relative to unexposed adult microglia. *P* values are calculated using the student t-test on the Delta C_T_. Bolded values reached our criteria of statistical significance where the FC is greater or less than 2-fold with a *P* value ≤ 5.0E-02

Exposure to these three TLR agonists reduced the expression of several inflammatory genes and receptors in adult microglia. There were 8 genes significantly decreased that were shared among all groups of adult microglia exposed to TLR agonists. Surprisingly, the class A CpG-ODN 1585 reduced expression of the most inflammatory genes of those tested (Fig. 3B and Table 2). Heatmap and cluster analysis of all 84 mouse inflammatory cytokines and receptors effectively depicted the differences between exposure groups (Fig. 3C). Moreover, it emphasized the increase in inflammatory gene expression in adult microglia in response to LPS and the reduction in inflammatory gene expression in adult microglia exposed to CpG-ODN 1585, which grouped with unexposed cells in medium alone.

The expression data corroborated much of our protein quantitation data (Figs. 1 and 2 vs. Fig. 3 and Table 2). For example, *Ccl4*, *Ccl5*, and *Tnf-α* transcripts were highest with LPS exposure, modestly increased with IMQ exposure, and not increased with CpG-ODN 1585 exposure (Table 2). This was also the case with CCL4, CCL5, and TNF-α protein quantification in the medium by Bio-Plex (Fig. 1). Therefore, exposure of adult microglia to LPS or IMQ was significantly proinflammatory, but exposure of adult microglia to CpG-ODN 1585 was largely anti-inflammatory with reduced expression and release of most immune effectors.

### Alterations in inflammatory gene expression in brains of PLX5622-treated and untreated mice after acute exposure to TLR agonists

Studying adult microglia ex vivo in a controlled environment is an important tool, but studies have demonstrated that cultured microglial expression signatures and responses are different from those of freshly isolated microglia [39, 51]. The CNS is a complex tissue consisting of many different cell and sub cell-types. These complexities can make in vivo studies challenging and more difficult to interpret, but it is important to understand the contribution of microglial activation to the innate immune response in the brain. Therefore, to investigate the impact of microglia on neuroinflammation in vivo, we treated mice for 28 days with PLX5622 to ablate microglia in the brain. We then exposed PLX5622-treated and untreated mice to TLR agonists LPS, IMQ, and CpG-ODN 1585 by intraventricular inoculation. Six hours later, brains were collected and measured for changes in inflammatory gene expression within the CNS.

**Fig. 4:**
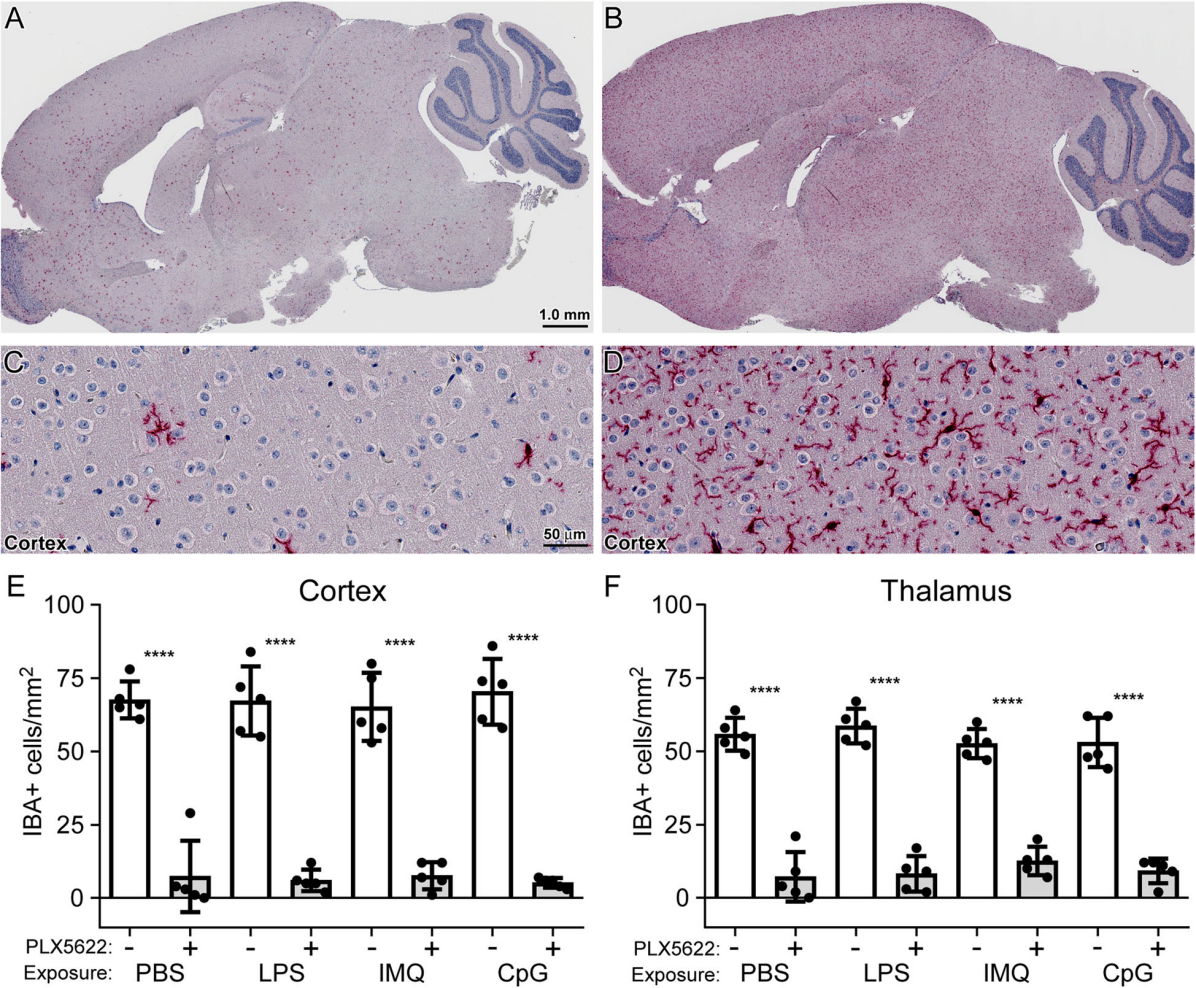
Determination of microglia in the adult mouse brain with and without PLX5622 treatment after acute TLR agonist or PBS exposure. Mice were either treated with PLX5622 (+) or untreated (−) for 28 days prior to a 6-h exposure to LPS, IMQ, CpG-ODN 1585 (CpG), or PBS as a control. Similar paraffin-embedded sections of cortex and thalamus were fixed and stained with antibody to Iba1. Panels **A**–**D** are representative images of a brain from a mouse either treated with PLX5622 (**A** and **C**) or untreated (**B** and **D**). IBA1-positive (IBA+) cells were enumerated by microscopic examination of the cortex (**E**) and thalamus (**F**) and reported as the number of positive cells per square millimeter. The white columns represent the mean number of IBA+ cells in untreated mice, and the gray columns represent the mean numbers of IBA+ cells in mice treated with PLX5622. Each dot represents an individual mouse. Error bars represent 1 standard deviation. Statistical analysis was performed using a Student’s *t* test on the mean IBA+ cells per mm^2^ to compare PLX5622-treated (reduced microglia) to untreated mice. ****P ≤ 0.0001

**Fig. 5:**
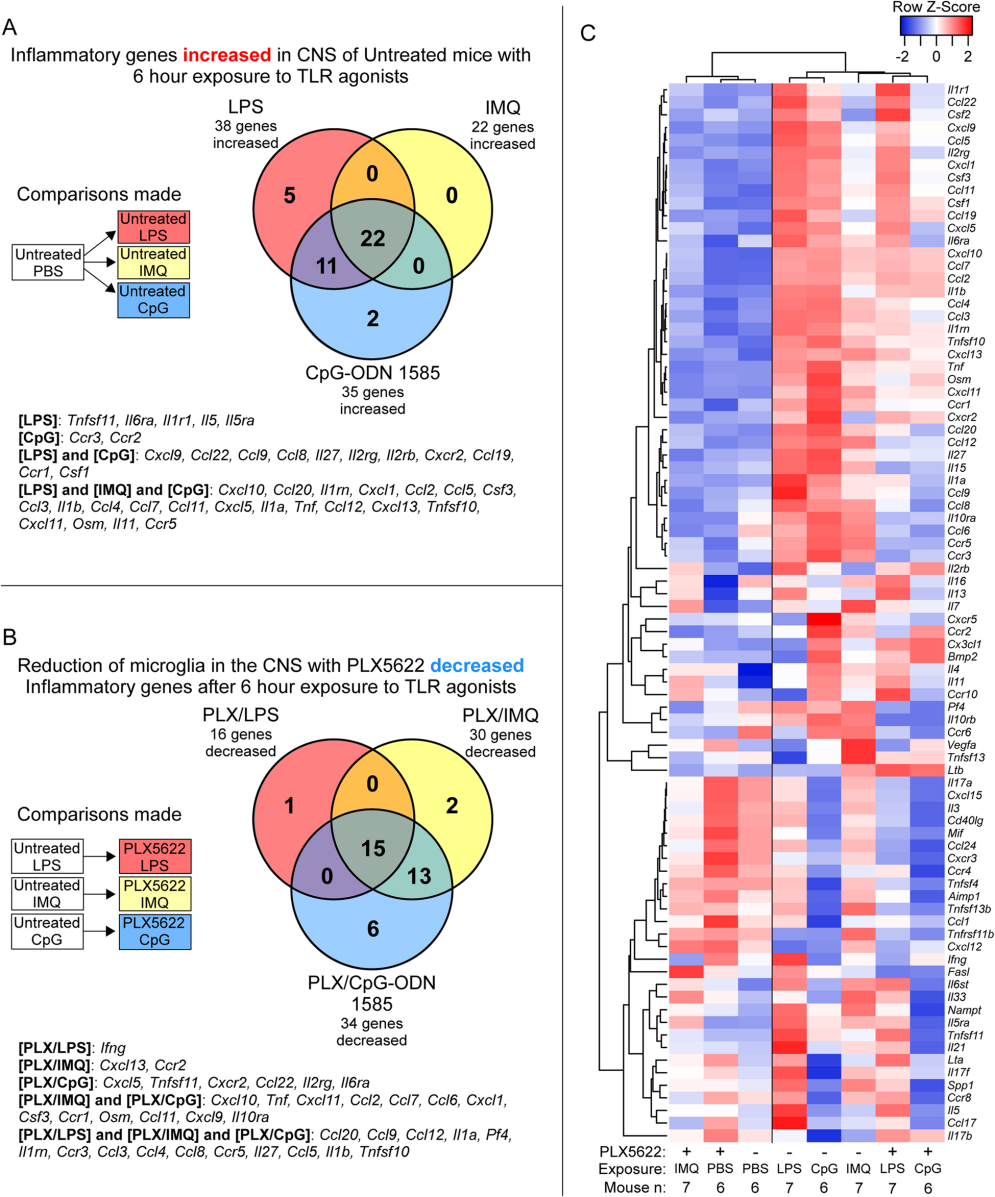
Alterations in expression of 84 inflammation-associated genes in adult mouse brain with and without PLX5622 treatment after acute TLR agonist or PBS exposure. Mice were either treated with PLX5622 or untreated for 28 days prior to a 6-h exposure to LPS, IMQ, CpG-ODN 1585 (CpG), or PBS as a control. Bulk RNA from brain of adult mice exposed to TLR agonists or PBS was assayed by qRT-PCR using the mouse inflammatory cytokine and receptor array. Panel **A** shows a Venn diagram of inflammatory genes statistically increased in untreated adult mice after TLR agonists exposure relative to mice exposed to PBS alone. Panel **B** displays a Venn diagram of inflammatory genes decreased when adult mice that were treated with PLX5622 (reduced microglia) then exposed to TLR agonists relative to untreated mice exposed to the same TLR agonists. Panel **C** is a Pearson correlation heatmap and average linkage hierarchical cluster analysis of the row Z-scores of the average Delta C_T_ from the array of 84 inflammatory cytokine and receptor genes obtained from the analysis of RNA from all adult mice exposed to TLR agonists and PBS alone. Mouse numbers in this analysis (*n*): PLX5622 treated mice exposed to LPS (*n* = 7), IMQ (*n* = 7), CpG-ODN 1585 (*n* = 6), PBS (*n* = 6); Untreated mice exposed to LPS (*n* = 7), IMQ (*n* = 7), CpG-ODN 1585 (*n* = 6), PBS (*n* = 6)

PLX5622 significantly reduced the number of microglia (IBA positive cells) in all treated cohorts to an average of 10% and 17% in the cortex and thalamus, respectively, compared to untreated mice (Fig. 4). Like the ex vivo studies performed above with adult microglia, we determined changes in gene expression by qRT-PCR array targeting 84 mouse inflammatory cytokine and receptor genes. We first verified that introduction of the TLR agonists LPS, IMQ, and CpG-ODN 1585 into untreated mice (without PLX5622 or microglia ablation) elicited a measurable TLR-mediated change in inflammatory transcripts compared to mice similarly injected with PBS as a control (refer to Additional file 2 for complete dataset). The top 20 inflammatory genes increased in response to all 3 TLR agonists are shown in Table 3. The greatest in fold change was *Cxcl10* for all agonists, reaching as high as ~ 648-fold higher in brains exposed to CpG-ODN 1585. Of the genes that were significantly increased, 22 were shared among the TLR agonist groups (Fig. 5A). Thus, a 6-h exposure was able to induce a vigorous response in the CNS that was easily measurable under our experimental procedure.

**Table 3:** Top 20 proinflammatory genes significantly increased in the brain of untreated mice after 6 h of exposure to TLR agonists relative to PBS-treated control mice (*n* = 6)

LPS (*n* = 7)			IMQ (*n* = 7)			CpG-ODN 1585 (*n* = 6)		
Gene	FC	*P* value	Gene	FC	*P* value	Gene	FC	*P* value
*Cxcl10*	545.7	2.9E-09	*Cxcl10*	203.4	4.0E-10	*Cxcl10*	648.2	< 1.0E-10
*Ccl20*	189.7	3.3E-07	*Ccl20*	39.4	1.9E-04	*Ccl20*	383.3	< 1.0E-10
*Il1rn*	157.3	6.0E-07	*Ccl2*	33.0	7.0E-05	*Il1rn*	115.6	4.0E-07
*Cxcl1*	95.2	4.9E-06	*Ccl7*	29.1	1.9E-05	*Ccl2*	103.3	4.5E-07
*Ccl2*	91.2	1.4E-05	*Il1rn*	25.1	1.9E-05	*Ccl7*	68.2	3.6E-07
*Ccl5*	82.7	7.7E-08	*Ccl4*	19.7	4.8E-05	*Il1b*	66.1	4.3E-06
*Csf3*	79.9	2.2E-08	*Ccl3*	18.9	1.4E-07	*Ccl4*	64.4	1.8E-06
*Ccl3*	64.6	5.5E-08	*Ccl12*	16.8	1.1E-05	*Ccl3*	59.3	8.2E-09
*Il1b*	58.1	4.3E-06	*Tnf*	9.6	5.5E-05	*Tnf*	52.1	1.6E-07
*Ccl4*	55.4	5.8E-06	*Ccl11*	9.6	6.4E-04	*Csf3*	47.0	5.8E-08
*Ccl7*	52.5	6.3E-05	*Cxcl11*	9.1	2.7E-03	*Cxcl1*	46.5	2.9E-06
*Ccl11*	43.0	2.7E-05	*Cxcl13*	8.5	3.6E-04	*Ccl5*	41.5	9.0E-10
*Cxcl5*	27.7	7.9E-04	*Ccl5*	7.8	2.6E-06	*Ccl11*	26.1	2.1E-05
*Il1a*	25.3	1.5E-06	*Csf3*	7.6	1.0E-03	*Ccl12*	21.0	4.4E-07
*Tnf*	23.8	5.8E-04	*Cxcl1*	6.8	1.7E-02	*Cxcl5*	20.8	8.1E-05
*Cxcl9*	17.7	8.2E-05	*Il1b*	6.7	1.6E-02	*Cxcl11*	18.9	1.7E-05
*Ccl22*	16.7	1.6E-05	*Cxcl5*	6.1	2.8E-02	*Cxcr2*	16.9	1.5E-04
*Ccl12*	16.4	1.7E-04	*Tnfsf10*	5.2	1.8E-06	*Cxcl9*	10.8	1.6E-08
*Ccl9*	15.5	7.9E-04	*Il1a*	3.9	8.3E-04	*Il1a*	10.1	7.5E-08
*Tnfsf11*	9.0	1.2E-03	*Ccr5*	3.6	1.2E-02	*Tnfsf10*	9.5	4.4E-08

*n* is the number of independent mouse RNA samples tested. Fold change (FC) relative to PBS-treated mice. *P* values are calculated using the student t-test on the Delta C_T_. All changes reached our criteria of statistical significance where the FC is greater than 2-fold with a *P* value ≤ 5.0E-02

We next analyzed the effect of reducing microglia with PLX5622 on gene expression when mice were similarly exposed to TLR agonists LPS, IMQ, and CpG-ODN 1585 (refer to Additional file 3 for complete dataset). Using the same array, we compared PLX5622-treated mice to untreated mice that were intraventricularly inoculated with either PBS or TLR agonists. To begin, we compared PLX5622-treated to untreated mice inoculated with PBS and saw only 1 out of the 84 inflammatory genes significantly changed (*IL11*, refer to Additional file 3). Thus, PLX5622 treatment alone for 28 days did not induce an overall change in the expression of genes associated with inflammation, as we have previously reported for longer PLX5622 treatments [29, 30].

Establishing that microglial ablation did not greatly affect brain inflammation in mice in general, we subsequently compared PLX5622-treated mice to untreated mice exposed to different TLR agonists to determine which genes in the brain were affected by the reduction of microglia. As one might expect, we observed a decrease in expression of many proinflammatory genes in PLX5622-treated mice relative to similarly inoculated untreated control mice (Table 4). PLX5622-treated mice exposed to CpG-ODN 1585 had the most genes significantly decreased followed by PLX5622-treated mice exposed to IMQ (Fig. 5B). A core group of 15 genes were decreased in all PLX5622-treated cohorts, indicating shared inflammatory genes whose expression was influenced by the presence of microglia in response to multiple TLR stimuli. These included genes predominantly expressed by microglia [10] in the healthy CNS (i.e., *Ccl9*, *IL1a*, and *IL1b*) (Fig. 5B and Table 4).

**Table 4:** Proinflammatory genes significantly decreased in the brain of PLX5622-treated relative to untreated mice after 6 h of exposure to TLR agonists

PLX5622-treated (*n* = 7) relative to Untreated exposed to LPS (*n* = 7)			PLX5622-treated (*n* = 7) relative to Untreated exposed to IMQ (*n* = 7)			PLX5622-treated (*n* = 6) relative to Untreated exposed to CpG-ODN 1585 (*n* = 6)		
Gene	FC	*P* value	Gene	FC	*P* value	Gene	FC	*P* value
*Ccl20*	− 11.1	5.1E-03	*Cxcl10*	− 18.6	9.9E-05	*Ccl20*	− 160.8	4.4E-06
*Ccl9*	− 8.8	6.8E-05	*Ccl3*	− 15.1	4.4E-06	*Ccl3*	− 14.1	7.5E-07
*Ccl12*	− 7.4	3.1E-04	*Tnf*	− 14.2	2.9E-04	*Ccl8*	− 9.4	7.4E-05
*Il1a*	− 6.5	3.7E-04	*Ccl20*	− 12.9	4.8E-03	*Ccl4*	− 9.1	1.1E-06
*Pf4*	− 6.2	8.9E-06	*Ccl4*	− 10.4	2.6E-06	*Ccr5*	− 9.0	8.1E-05
*Il1rn*	− 5.1	3.7E-04	*Ccl8*	− 9.6	5.0E-04	*Il1a*	− 8.1	4.9E-06
*Ccr3*	− 5.1	2.1E-03	*Cxcl11*	− 9.4	5.6E-04	*Ccl12*	− 8.0	3.9E-06
*Ccl3*	− 5.1	3.2E-04	*Pf4*	− 9.2	5.0E-06	*Cxcl5*	− 7.7	1.8E-05
*Ccl4*	− 5.1	7.5E-04	*Ccl12*	− 9.0	5.1E-05	*Ccl9*	− 6.9	1.4E-04
*Ccl8*	− 5.0	1.2E-02	*Ccl2*	− 8.1	1.3E-03	*Tnf*	− 6.8	7.1E-06
*Ccr5*	− 4.9	1.0E-04	*Il1rn*	− 7.6	6.0E-05	*Pf4*	− 6.5	7.3E-06
*Il27*	− 3.2	3.3E-02	*Ccl7*	− 6.9	2.9E-04	*Ccr3*	− 6.2	3.6E-04
*Ccl5*	− 3.0	6.1E-03	*Cxcl13*	− 6.0	5.5E-03	*Csf3*	− 6.1	3.4E-07
*Il1b*	− 2.6	3.2E-02	*Ccr5*	− 5.3	3.3E-05	*Il1rn*	− 6.0	1.2E-05
*Ifng*	− 2.2	4.5E-02	*Ccl9*	− 4.8	2.4E-03	*Ccl5*	− 5.5	1.3E-06
*Tnfsf10*	− 2.1	1.5E-02	*Il1b*	− 4.7	2.4E-02	*Cxcl1*	− 5.4	5.9E-04
			*Ccl6*	− 4.7	1.4E-04	*Tnfsf11*	− 5.2	3.7E-03
			*Ccl5*	− 4.4	8.6E-05	*Ccl6*	− 5.2	1.8E-04
			*Il1a*	− 4.0	5.8E-03	*Ccr1*	− 4.5	1.8E-04
			*Ccr2*	− 4.0	2.0E-02	*Cxcr2*	− 4.4	2.5E-03
			*Cxcl1*	− 3.9	5.1E-02	*Il27*	− 4.3	1.2E-02
			*Csf3*	− 3.6	9.0E-03	*Cxcl11*	− 4.1	3.2E-04
			*Ccr1*	− 3.5	6.8E-03	*Ccl22*	− 3.4	1.6E-06
			*Il27*	− 3.4	5.4E-03	*Ccl11*	− 3.3	2.6E-05
			*Ccr3*	− 3.4	1.6E-04	*Cxcl10*	− 3.3	1.7E-06
			*Osm*	− 3.3	1.2E-02	*Cxcl9*	− 3.3	2.8E-02
			*Tnfsf10*	− 3.2	2.9E-03	*Il2rg*	− 2.9	3.9E-05
			*Ccl11*	− 3.2	8.8E-04	*Il1b*	− 2.8	8.7E-03
			*Cxcl9*	− 2.7	3.7E-02	*Osm*	− 2.7	1.1E-02
			*Il10ra*	− 2.3	1.9E-03	*Tnfsf10*	− 2.6	8.7E-06
						*Ccl7*	− 2.4	5.7E-05
						*Il6ra*	− 2.2	5.6E-04
						*Il10ra*	− 2.1	2.8E-03
						*Ccl2*	− 2.0	1.3E-03

*n* is the number of independent mouse RNA samples tested. FC is fold change in gene expression as indicated. *P* values are calculated using the student *t* test on the Delta C_T_. All changes reached our criteria of statistical significance where the FC is less than 2.0-fold with a *P* value ≤ 5.0E-02

Though loss of microglia in the CNS diminished the expression of many genes induced by all three TLR agonists, there were several inflammatory genes whose expression was increased in untreated mice (Table 3) that were not decreased in mice with reduced microglia that were exposed to TLR agonists (refer to Additional file 4 for comparison). Expression of over twenty genes (i.e. *Cxcl10*, *Cxcl13*, *Ccl7*, and *Ccl11*) was not significantly decreased in PLX5622-treated relative to untreated mice after exposure to LPS. Likewise, several genes (i.e., *Cxcl13*, *Csf1*, and *Ccr2*) were equally induced in PLX5622-treated and untreated mice stimulated with CpG-ODN 1585. Interestingly, only 2 of the genes assayed (*Cxcl5* and *IL11*) were equally increased in PLX5622-treated and untreated mice stimulated with IMQ. This suggested that expression of numerous genes in response to LPS or CpG might be independent or only partially dependent on microglial influences, and that the CNS might have an effective compensatory mechanism for some stimuli when microglia are reduced.

A heatmap hierarchical cluster analysis of the changes in gene expression to TLR stimulation and PLX5622 treatment indicated reduction of microglia affected the response to IMQ the most (Fig. 5C). PLX5622-treated IMQ-stimulated mice grouped closely with PLX5622-treated and untreated mice exposed to PBS. This analysis further supported our observation that exposure of mice to LPS and CpG-ODN 1585 induced a similar subset of inflammatory genes, since these cohorts grouped tightly. Thus, the innate immune response to TLR4 agonist LPS and TLR9 agonist CpG-ODN 1585 was only partially affected by the reduction in microglia in the adult mouse brain, but loss of microglia in the CNS more severely dysregulated innate immune responses to IMQ (TLR7) stimulation. Furthermore, though cultured microglia exposure to CpG-ODN 1585 was anti-inflammatory (Table 2 and Fig. 3), the introduction of this TLR9 agonist into the brain had a proinflammatory effect (Table 3 and Fig. 5).

### Alterations in expression of genes associated with TLR pathway signaling in brains of PLX5622-treated and untreated mice after acute exposure to TLR agonists

**Fig. 6:**
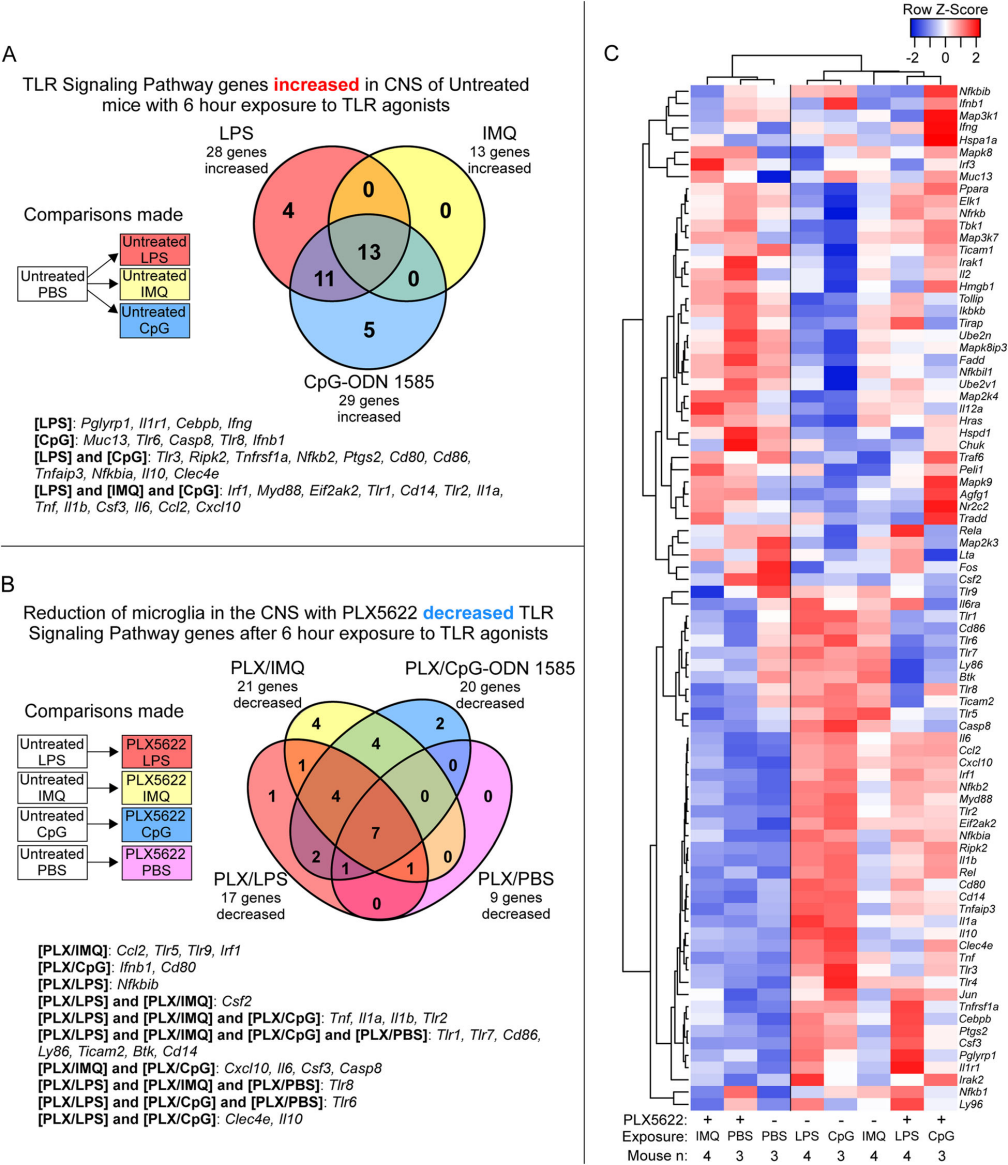
Alterations in expression of 84 TLR signaling pathway-associated genes in adult mouse brain with and without PLX5622 treatment after acute TLR agonist or PBS exposure. Mice were either treated with PLX5622 or untreated for 28 days prior to a 6-h exposure to LPS, IMQ, CpG-ODN 1585 (CpG), or PBS as a control. Bulk RNA from brain of adult mice exposed to TLR agonists or PBS was assayed by qRT-PCR using the mouse Toll-like receptor signaling pathway array. Panel **A** presents a Venn diagram of TLR signaling-associated genes statistically increased in untreated adult mice after TLR agonists exposure relative to mice exposed to PBS alone. Panel **B** displays a Venn diagram of TLR signaling-associated genes decreased when adult mice that were treated with PLX5622 (reduced microglia) then exposed to TLR agonists relative to untreated mice exposed to the same TLR agonists. Panel **C** is a Pearson correlation heatmap and average linkage hierarchical cluster analysis of the row Z-scores of the average Delta CT from the array of 84 TLR signaling-associated genes obtained from the analysis of RNA from all adult mice exposed to TLR agonists and PBS alone. Mouse numbers in this analysis (*n*): PLX5622 treated mice exposed to LPS (*n* = 4), IMQ (*n* = 4), CpG-ODN 1585 (*n* = 3), PBS (*n* = 3); Untreated mice exposed to LPS (*n* = 4), IMQ (*n* = 4), CpG-ODN 1585 (*n* = 3), PBS (*n* = 3)

With reduction of microglia having varying effects on inflammatory responses when mice were exposed to LPS, CpG-ODN 1585, or IMQ, we asked which aspects to TLR signaling were influenced by microglia in the brain. Utilizing the qRT-PCR array by QIAGEN that targets 84 genes associated with the TLR signaling pathway, we first assessed the changes in gene expression in the brains of untreated mice (without PLX5622 or microglia ablation) exposed to LPS, IMQ, or CpG-ODN 1585 relative to PBS controls (refer to Additional file 5). Exposure to LPS and CpG-ODN 1585 had a similar number of genes increased (28 and 29, respectively) and shared 24 genes associated with the TLR signaling pathway (Table 5 and Fig. 6A). A core group of 13 increased genes were shared among all groups exposed to TLR agonists. Stimulation with all agonists reduced expression of *Csf2* in the brain by 43- to 83-fold (Table 5). Thus, untreated mice acutely exposed for 6 h to LPS and CpG-ODN 1585 demonstrated more similarities in their overall TLR signaling pathway responses, including increases in genes associated with the NF-κB pathway (*Nfkb2*, *Nfkbia*, *Tnfaip3*, *Tnfrsf1a*, and *IL10*).

**Table 5:** TLR signaling pathway associated genes significantly altered in the brain of untreated mice after 6 h of exposure to TLR agonists relative to PBS-treated control mice (*n* = 3)

LPS (*n* = 4)			IMQ (*n* = 4)			CpG-ODN 1585 (*n* = 3)		
Gene	FC	*P* value	Gene	FC	*P* value	Gene	FC	*P* value
*Cxcl10*	500.5	< 1.0E-10	*Cxcl10*	182.7	< 1.0E-10	*Cxcl10*	873.3	< 1.0E-10
*Ccl2*	110.3	< 1.0E-10	*Ccl2*	28.6	< 1.0E-10	*Ccl2*	182.9	< 1.0E-10
*Il6*	65.8	< 1.0E-10	*Tnf*	14.0	< 1.0E-10	*Il6*	108.0	< 1.0E-10
*Csf3*	51.2	< 1.0E-10	*Il6*	10.8	< 1.0E-10	*Tnf*	72.9	< 1.0E-10
*Il1b*	49.5	< 1.0E-10	*Tlr2*	4.2	2.0E-07	*Il1b*	69.7	< 1.0E-10
*Tnf*	42.0	< 1.0E-10	*Il1b*	3.8	1.1E-06	*Clec4e*	68.2	< 1.0E-10
*Clec4e*	29.0	< 1.0E-10	*Eif2ak2*	3.3	1.4E-05	*Csf3*	32.5	< 1.0E-10
*Il1a*	17.1	< 1.0E-10	*Irf1*	2.6	4.0E-04	*Tlr2*	21.0	< 1.0E-10
*Tlr2*	16.4	< 1.0E-10	*Csf3*	2.6	4.8E-04	*Cd14*	12.6	< 1.0E-10
*Cd14*	11.9	< 1.0E-10	*Tlr1*	2.5	6.4E-04	*Il1a*	10.6	< 1.0E-10
*Tlr1*	7.1	< 1.0E-10	*Il1a*	2.2	3.3E-03	*Il10*	7.6	2.1E-09
*Il10*	6.8	< 1.0E-10	*Myd88*	2.1	5.8E-03	*Tlr1*	6.9	1.1E-08
*Nfkbia*	5.5	1.5E-09	*Cd14*	2.1	5.8E-03	*Eif2ak2*	6.0	9.1E-08
*Eif2ak2*	4.3	2.1E-07	*Ifnb1*	− 4.4	7.9E-08	*Irf1*	5.8	1.7E-07
*Myd88*	4.1	4.6E-07	*Csf2*	− 82.5	< 1.0E-10	*Ifnb1*	5.5	3.7E-07
*Tnfaip3*	4.0	8.6E-07				*Myd88*	4.9	1.9E-06
*Irf1*	3.8	1.4E-06				*Nfkbia*	4.4	8.0E-06
*Cd86*	3.7	2.3E-06				*Tnfaip3*	3.7	8.2E-05
*Cd80*	3.1	3.8E-05				*Tlr3*	3.0	8.9E-04
*Nfkb2*	2.7	3.1E-04				*Tlr8*	3.0	9.6E-04
*Ptgs2*	2.7	3.1E-04				*Cd80*	2.8	2.0E-03
*Ifng*	2.6	7.2E-04				*Nfkb2*	2.8	2.1E-03
*Tnfrsf1a*	2.4	1.5E-03				*Cd86*	2.6	4.7E-03
*Ripk2*	2.3	2.0E-03				*Ripk2*	2.5	5.7E-03
*Cebpb*	2.3	2.8E-03				*Casp8*	2.5	5.7E-03
*Il1r1*	2.1	7.2E-03				*Tnfrsf1a*	2.5	6.4E-03
*Tlr3*	2.1	6.7E-03				*Ptgs2*	2.4	7.3E-03
*Pglyrp1*	2.0	9.7E-03				*Tlr6*	2.3	1.0E-02
*Fos*	− 2.0	9.7E-03				*Muc13*	2.0	3.1E-02
*Ifnb1*	− 3.1	4.7E-05				*Nfkbil1*	− 2.1	2.5E-02
*Csf2*	− 49.4	< 1.0E-10				*Csf2*	− 43.7	< 1.0E-10

*n* is the number of independent mouse RNA samples tested. Fold change (FC) relative to PBS treated mice. *P* values are calculated using the student t-test on the Delta CT. All changes reached our criteria of statistical significance where the FC is greater or less than 2-fold with a *P* value ≤ 5.0E-02

**Fig. 7:**
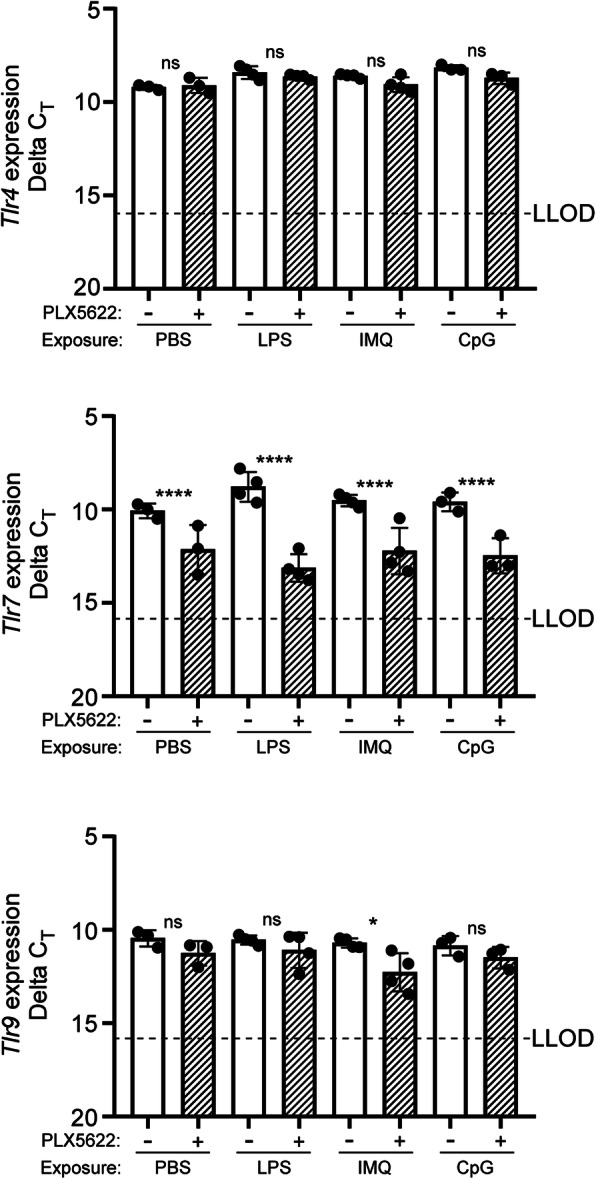
Transcription of genes encoding TLR4, TLR7, and TLR9 in adult mouse brain with and without PLX5622 treatment after acute TLR agonist or PBS exposure. Expression of *Tlr4*, *Tlr7*, and *Tlr9* was measured by qRT-PCR in mouse brains either treated with PLX5622 (+) or untreated (−) for 28 days prior to a 6-h exposure to LPS, IMQ, CpG-ODN 1585 (CpG), or PBS as a control. The Delta C_T_ was plotted for each cohort where each dot represents a single mouse assayed. *LLOD* lower limit of detection of transcript based on a C_T_ cutoff value of 35. Statistical analysis was performed using a Student’s *t* test on the mean Delta C_T_ to compare PLX5622-treated (reduced microglia) to untreated mice. ****P ≤ 0.0001; *P ≤ 0.05, *ns* not significant

Next, we used the TLR signaling pathway array to evaluate the influence of microglia on TLR signaling in the CNS comparing PLX5622-treated to untreated mice that were exposed to TLR agonists (refer to Additional file 6 for complete dataset). Comparing PLX5622-treated to untreated mice injected with PBS alone indicated 1 gene was increased (*Ifng*) and 9 genes were decreased with PLX5622 treatment alone (Table 6). Most of the decreased genes (i.e., *Cd14*, *Cd86*, *Ly86*, and *Ticam2*) are predominantly expressed by microglia in the healthy brain [10], so their reduction by PLX5622 treatment was not unexpected. Of interest was the notable decrease in expression of *Tlr7*, the receptor for IMQ, in all PLX5622-treated mice, but surprisingly we did not detect an appreciable reduction in *Tlr4* or *Tlr9* expression in PLX5622-treated mice with reduced microglia (Fig. 7).

**Table 6:** TLR signaling pathway associated genes altered in the brain of PLX5622-treated relative to Untreated mice after 6 hours of exposure to TLR agonists or PBS

	PLX5622-treated (*n* = 3) relative to Untreated exposed to PBS (*n* = 3)		PLX5622-treated (*n* = 4) relative to Untreated exposed to LPS (*n* = 4)		PLX5622-treated (*n* = 4) relative to Untreated exposed to IMQ (*n* = 4)		PLX5622-treated (*n* = 3) relative to Untreated exposed to CpG-ODN 1585 (*n* = 3)	
Gene	FC	*P* value	FC	*P* value	FC	*P* value	FC	*P* value
*Btk*	− **2.2**	**8.5E-03**	− **4.2**	**5.2E-07**	− **3.0**	**2.8E-04**	− **2.3**	**1.1E-02**
*Casp8*	− 1.3	3.5E-01	− 1.8	4.6E-02	− **2.0**	**2.0E-02**	− **2.9**	**1.0E-03**
*Ccl2*	− 1.4	2.6E-01	− 1.0	9.8E-01	− **8.0**	< **1.1E-10**	− 1.6	1.6E-01
*Cd14*	− **2.6**	**1.4E-03**	− **3.0**	**1.2E-04**	− **3.9**	**8.9E-06**	− **4.1**	**1.6E-05**
*Cd80*	− 1.5	2.1E-01	− 1.4	2.4E-01	− 1.4	2.6E-01	− **2.2**	**1.9E-02**
*Cd86*	− **6.1**	**4.9E-09**	− **13.6**	< **1.1E-10**	− **5.1**	**9.7E-08**	− **11.0**	< **1.1E-10**
*Clec4e*	1.3	4.0E-01	− **20.5**	< **1.1E-10**	− 1.3	4.2E-01	− **3.4**	**1.6E-04**
*Csf2*	− 1.7	6.6E-02	**2.3**	**4.4E-03**	**2.2**	**1.0E-02**	− 1.5	2.5E-01
*Csf3*	1.2	5.3E-01	1.6	8.3E-02	− **2.4**	**3.9E-03**	− **3.9**	**3.4E-05**
*Cxcl10*	1.8	6.0E-02	− 1.4	2.8E-01	− **13.2**	< **1.1E-10**	− **2.6**	**3.4E-03**
*Ifnb1*	1.2	5.0E-01	1.2	5.3E-01	1.3	3.6E-01	− **2.2**	**1.4E-02**
*Ifng*	**2.5**	**3.0E-03**	1.2	5.6E-01	1.0	9.8E-01	**4.4**	**6.8E-06**
*Il10*	1.2	4.8E-01	− **4.0**	**1.5E-06**	1.1	8.7E-01	− **3.2**	**4.3E-04**
*Il1a*	− 1.5	2.1E-01	− **5.8**	**1.1E-09**	− **2.9**	**5.1E-04**	− **7.0**	**4.3E-09**
*Il1b*	− 1.1	6.6E-01	− **4.1**	**7.5E-07**	− **2.9**	**5.1E-04**	− **2.1**	**2.0E-02**
*Il6*	− 1.6	1.2E-01	− 1.6	8.8E-02	− **5.2**	**7.6E-08**	− **2.0**	**3.2E-02**
*Irf1*	1.3	4.2E-01	1.2	4.9E-01	− **2.0**	**2.4E-02**	− 1.6	1.3E-01
*Ly86*	− **3.5**	**4.8E-05**	− **10.7**	< **1.1E-10**	− **3.2**	**1.5E-04**	− **4.3**	**1.0E-05**
*Nfkbib*	1.2	6.5E-01	− **2.0**	**1.6E-02**	− 1.1	8.2E-01	1.5	1.9E-01
*Ticam2*	− **3.8**	**1.2E-05**	− **8.7**	< **1.1E-10**	− **5.5**	**2.5E-08**	− **2.7**	**2.7E-03**
*Tlr1*	− **7.4**	**1.0E-10**	− **28.1**	< **1.1E-10**	− **7.3**	< **1.1E-10**	− **12.0**	< **1.1E-10**
*Tlr2*	1.2	6.3E-01	− **2.1**	**8.6E-03**	− **3.4**	**5.3E-05**	− **3.2**	**4.0E-04**
*Tlr5*	− 1.3	4.5E-01	− 1.3	4.2E-01	− **2.9**	**4.3E-04**	− 1.8	6.7E-02
*Tlr6*	− **3.4**	**5.8E-05**	− **5.2**	**1.1E-08**	− 1.7	9.8E-02	− **5.4**	**3.5E-07**
*Tlr7*	− **4.3**	**2.1E-06**	− **17.1**	< **1.1E-10**	− **5.6**	**1.7E-08**	− **6.8**	**6.2E-09**
*Tlr8*	− **4.6**	**7.6E-07**	− **13.5**	< **1.1E-10**	− **12.6**	< **1.1E-10**	− 1.7	1.2E-01
*Tlr9*	− 1.8	6.3E-02	− 1.2	4.8E-01	− **2.6**	**1.7E-03**	− 1.4	2.6E-01
*Tnf*	1.6	1.1E-01	− **10.1**	< **1.1E-10**	− **11.4**	< **1.1E-10**	− **4.5**	**4.6E-06**

*n* is the number of independent mouse RNA samples tested. FC is fold change in gene expression as indicated. *P* values are calculated using the student t-test on the Delta C_T_. All changes in bold reached our criteria of statistical significance where FC is greater or less than 2.0-fold with a *P* value ≤ 5.0E-02

When contrasting PLX5622-treated to untreated mice exposed to LPS, we observed decreases in 17 genes associated with TLR signaling (Table 6 and Fig. 6B). Besides the genes decreased with PLX5622 treatment alone, we also noted reduced expression of genes encoding cytokines (*IL1b* and *Tnf*), receptors (*Clec4e* and *Tlr2*), and an NF-κB component (*Nfkbib*) associated with TLR signaling (refer to Additional file 6). More interesting were genes normally increased with LPS introduction in the CNS that were unchanged in the brains of mice with reduced microglia also exposed to LPS (refer to Additional file 7 for comparison). These included genes involved in cytokine signaling (*Cebpb*, *Csf3*, *Ifng*, *IL6*, and *Ccl2*), TLR interacting/adaptor proteins (*Myd88*, *Pglyrp1*, and *Ripk2*), interferon regulatory factor (IRF) signaling (*Cxcl10*, *Ifnb1*, *Ifng*, and *Irf1*), and NF-κB signaling (*Nfkb1*, *Nfkb2*, and *Tnfaip3*). Thus, reduction of microglia in the CNS decreased a subset of genes associated with TLR4 signaling and microglia, but many downstream signaling systems were unaffected in PLX5622-treated mice exposed to LPS.

Comparing PLX5622-treated to untreated mice exposed to IMQ, 21 genes were reduced associated with TLR signaling (Table 6 and Fig. 6B). Along with the reductions seen with PLX5622 treatment alone, we observed further reductions in several additional TLR receptor transcripts (*Tlr2*, *Tlr5*, and *Tlr9*), JAK/STAT signaling associated genes (*Ccl2*, *Csf2*, and *IL6*), and IRF signaling (*Cxcl10* and *Irf1*) (refer to Additional file 6). Only three genes (*Eif2ak2*, *Ifnb1*, and *Myd88*) that were normally altered in the CNS with IMQ exposure were unaffected by the reduction in microglia (refer to Additional file 7). Thus, reducing microglia in the CNS had a disruptive effect on responses to the TLR7 agonist IMQ, which led to reduced expression of key genes involved in several downstream TLR-associated signaling pathways. This dysregulation was likely due in large part to the large reduction in *Tlr7* expression in all PLX5622-treated mice (Fig. 7 and Table 6).

Lastly, we assessed changes in gene expression associated with TLR signaling in PLX5622-treated relative to untreated mice exposed to CpG-ODN 1585 (refer to Additional file 6). Twenty TLR signaling-associated genes were decreased and 1 gene was increased (*Ifng*) in PLX5622-treated mice (Table 6 and Fig. 6B). Adding to the transcripts reduced with PLX5622 treatment alone, additional decreases in expression were noted for genes associated with NF-κB signaling (*Casp8*, *IL10*, *IL1b*, and *Tnf*), IRF signaling (*Cxcl10* and *Ifnb1*), and the fungal/parasitic response (*Clec4e* and *Tlr2*). Again, the reduction of microglia did not decrease expression of several genes that are normally increased in the CNS with CpG-ODN 1585 exposure. These included genes associated with NF-κB signaling (*Nfkb2* and *Tnfaip3*), TLR interacting/adaptor proteins (*Myd88* and *Ripk2*), and pathogen-specific responses (*Ccl2*, *Ptgs2*, *Ripk2*, *Tnfrsf1a*, *Eif2ak2*, and *Tlr3*) (refer to Additional file 7).

A heatmap hierarchical cluster analysis of the changes in TLR signaling pathway gene expression in response to TLR agonist stimulation and PLX5622 treatment indicated the reduction of microglia affected the overall response to IMQ the most, since these mice grouped with PBS exposed control mice (Fig. 6C). Thus, when microglia were reduced in the CNS the overall dysregulation was more pronounced in PLX5622-treated mice exposed to IMQ, suggesting the reduction of microglia in the CNS had a greater effect on the response to IMQ relative to LPS or CpG-ODN 1585.

## Discussion

In our present study, we compared the innate immune response in highly purified adult microglia and neonatal (P3) microglia. Both microglia isolates were similarly cultured under conditions that reduce activation to maintain cells in a homeostatic state, allowing us to assess and better identify the effects of different TLR agonists on key immune-related protein/gene expression. There are many analogous past studies evaluating the innate immune response of neonatal microglia under a variety of culturing conditions [9, 33, 52, 53], but several reports underscore the importance of media supplements and developmental stage on microglia health and responsiveness [39, 54–56]. Postnatal microglia (P1 to P30) differ broadly in their expression signatures compared to adult microglia and demonstrate decreased expression of numerous surface receptors including TLR3, TLR7, and TLR9 [12, 39]. Furthermore, freshly isolated postnatal microglia lack the expression signature of adult microglia until after 2 months of age [39].

We observed differences in TLR induced proinflammatory mediators and in the magnitude of the response between ex vivo homeostatic adult and P3 microglia. This is not surprising given the profound differences in gene expression between adult and P3 microglia. What was unexpected was the limited secretion of immune effectors in both microglia populations and the reduction in gene expression of inflammatory genes in adult microglia in response to exposure to the class A TLR9 agonist CpG-ODN 1585. Similar results have been reported with cultured murine cardiac fibroblasts and macrophages exposed to CpG-ODN 1585 [57], which differ from reports of proinflammatory responses in dendritic cells [57–59]. These studies demonstrate that in vitro responses to different classes of CpG-ODNs are cell-specific, and it appears that microglia are more like macrophages rather than dendritic cells in their response to CpG-ODN 1585.

It was evident that in most cases our changes in adult microglia inflammatory gene expression ex vivo was greater relative to reported expression profiles of postnatal microglia. For example, when exposed to TLR agonists, several important inflammatory transcripts including *Tnf*, *IL1b*, *Ccl2*, *Ccl5*, and *Cxcl10* were more elevated in our adult microglia samples relative to published postnatal microglia results [9, 33, 60, 61]. These observations largely correlated with our immune effector secretion results (Fig. 1), but one should be cautious in forming definitive conclusions due to experimental variations such as exposure times, microglial purity, and quantitation methodology. Interestingly, there were a few instances (i.e., CCL1, CCL11, CCL20, and CXCL13) where our adult microglia qRT-PCR results differed from our immune effector secretion findings. This was expected since RNA analysis offers only a “snapshot” in time of the gene expression profile, but the assessment of protein secretion in the media is cumulative over time.

Our studies exposing purified microglia to TLR agonists are not without their limitations. Though we have attempted to maintain microglia in a homeostatic state during culture [39], merely isolating microglia from the brain and introduction to culture media alters their activation state and expression profile [39, 51]. Furthermore, we cultured our microglia using FCS that has been shown to alter microglial phagocytic responses [51]. Ultimately, microglia are reliant on specific cues from the CNS microenvironment that allow these specialized cells to maintain the unadulterated homeostatic phenotype seen in the brain [39, 51, 62, 63], which cell culture cannot fully replicate.

Studying highly purified microglia ex vivo offers several obvious advantages, but microglia make up only ~ 15–20% of the cells in the CNS where they interact with other glia and neurons. As such, we sought to address the contribution of microglia on the overall innate immune response in the context of the complexities of the CNS. We found that reducing microglia in the CNS using CSF-1R inhibitor PLX5622 had the greatest effect on inflammatory gene expression in mice exposed to IMQ and the least effect in mice exposed to LPS. This would indicate that TLR7 responses in the CNS are more heavily reliant on the presence of microglia than TLR4 responses.

A similar result to LPS exposure and microglial influences on astrocytes was reported by Liddelow et al., where the authors treated mice with CSF-1R inhibitor PLX3397 (Pexidartinib, a similar compound to PLX5622) to reduce microglia and exposed them to LPS [7]. The LPS stimulated PLX3397-treated mice were still capable of inducing some inflammation and a partial A1-reactive astrocytic phenotype, albeit reduced. One interpretation is that the partial reduction of microglia in the CNS, which is a limitation of our studies as well, leaves enough microglia (just ~ 10%) to elicit an adequate global response to stimuli. Alternatively, the TLR4 response may be due to the presence of endothelial cells throughout the CNS. Microglia and endothelial cells naturally express near equivalent levels of *Tlr4* transcript in the murine CNS [10], which was unaffected with microglia ablation. Moreover, endothelial cells can express and secrete numerous immune effectors in response to insult and injury [64–66]. Thus, endothelial cells in the CNS may be a key source for several cytokines including the A1 astrocyte-inducing cytokines (TNF and IL-1a) in response to LPS in PLX5622-treated mice.

Single-cell sequencing of mouse brain suggests that *Tlr7* is expressed predominately by microglia in the adult murine CNS [10]. This might explain the dependence on adequate microglia numbers for a robust response to IMQ in the CNS. In our analysis, mice with reduced microglia in the CNS exposed to the TLR7 agonist IMQ grouped with control mice when expression profiles were compared (Figs. 6 and 7). Reducing microglia in the CNS with PLX5622 treatment correlated with a decrease in the overall expression of *Tlr7* between 4- to 17-fold. This decrease in receptor expression likely caused dysfunction in cellular signaling induced by IMQ, leading to a lack of *Irf1* expression that is an important transcription factor involved in downstream TLR signaling [67–71].

A perplexing aspect to our results is the partial response to TLR9 agonist CpG-ODN 1585 and the unchanged level of *Tlr9* expression with microglia reduction in CNS. Like other *Tlr* genes, *Tlr9* is expressed by microglia in the adult brain [9, 10], but in situ hybridization and immunostaining indicate that *Tlr9* expression is also associated with neurons in many brain regions in adult mice by 5 months of age [12]. Perhaps neurons in the adult CNS that adequately expresses *Tlr9* respond to CpG-ODN 1585 and partially compensate for the reduction of microglia in our studies, but this remains to be determined.

Lastly, we consider the contrasting results we obtained when CpG-ODN 1585 was introduced to microglia ex vivo or into the adult brain. Cultured microglia responded by reducing their expression of most inflammatory genes, but when introduced into the complex tissue of the adult brain, a robust proinflammatory response was induced that included several microglia-associated genes (i.e., *Tnf*, *IL1a*, *IL1b*, and *IL6*). Our in vivo results differed from those of Butchi et al., where inoculation of CpG-ODN 1585 in 2-day-old mice did not increase *Tnf* or *Ifnb1* expression [42]. This is likely explained by the low level of expression of *Tlr9* in the neonatal mouse brain relative to the adult [12], and the possibility that neonates lack the development to respond to this class A agonists. Furthermore, our ex vivo results are a staunch reminder that cellular responses in a monoculture system will likely differ from the responses of a tissue with cellular cross-communication.

## Conclusions

Our studies demonstrate that cultured adult and P3 microglia can autonomously respond to TLR agonists LPS and IMQ but lack the ability to respond to class A CpG-ODN 1585. Though ex vivo experimental approaches provide valuable insight into the functions of the different subpopulations in the CNS, the complexities of the cellular interactions within tissues cannot be replicated in monoculture. In vivo studies revealed a group of core genes associated with inflammation and TLR signaling that were increased in the CNS after introduction of all three TLR agonists. Moreover, the necessity for microglia in the CNS to mediate innate immune response varied depending on the stimuli, where TLR7-mediated innate immune responses were more dependent on the presence of microglia. Our studies also imply that other cell-types within the CNS can compensate when microglia are reduced, allowing for partial innate immune responses to TLR4 and TLR9 agonists.

## Supplementary Information

**Additional file 1.** Adult Microglia Inflammatory Cytokine and Receptor Array dataset.

**Additional file 2.** Inflammatory gene analysis of Untreated mice exposed to TLR agonists.

**Additional file 3.** Inflammatory gene analysis dataset_PLX5622 Treated with Untreated mice exposed to TLR agonists.

**Additional file 4.** Inflammatory genes increased in Untreated mice with TLR agonists but not decreased with microglia reduction.

**Additional file 5.** TLR signaling pathway gene analysis of Untreated mice exposed to TLR agonists.

**Additional file 6.** TLR signaling pathway gene analysis dataset_PLX5622 Treated to Untreated mice exposed to TLR agonists.

**Additional file 7.** TLR signaling pathway genes increased in Untreated mice with TLR agonists but not decreased with microglia reduction.

## Data Availability

All data generated or analyzed during this study are included in this published article and its supplementary information files.

## Abbreviations

CNS: Central nervous system
FCS: Fetal calf serum
TLR: Toll-like receptor
LPS: Lipopolysaccharide
IMQ: Imiquimod
CpG-ODN: CpG oligonucleotides
PAMPs: Pathogen-associated molecular patterns
DAMPs: Damage/danger-associated molecular patterns
CSF-1R: CSF-1 receptor
MACS: Magnetic-activated cell separation
